# A New Chaotic System with Stable Equilibrium: Entropy Analysis, Parameter Estimation, and Circuit Design

**DOI:** 10.3390/e20090670

**Published:** 2018-09-05

**Authors:** Tomasz Kapitaniak, S. Alireza Mohammadi, Saad Mekhilef, Fawaz E. Alsaadi, Tasawar Hayat, Viet-Thanh Pham

**Affiliations:** 1 Division of Dynamics, Lodz University of Technology, Stefanowskiego 1/15, 90-924 Lodz, Poland; 2 Power Electronics and Renewable Energy Research Laboratory (PEARL), Department of Electrical Engineering, Faculty of Engineering, University of Malaya, Kuala Lumpur 50603, Malaysia; 3 Department of Information Technology, Faculty of Computing and IT, King Abdulaziz University, Jeddah 21589, Saudi Arabia; 4 Department of Mathematics, Quaid-I-Azam University 45320, Islamabad 44000, Pakistan; 5 NAAM Research Group, King Abdulaziz University, Jeddah 21589, Saudi Arabia; 6 Modeling Evolutionary Algorithms Simulation and Artificial Intelligence, Faculty of Electrical & Electronics Engineering, Ton Duc Thang University, Ho Chi Minh City, Vietnam

**Keywords:** chaotic flow, hidden attractor, multistable, entropy

## Abstract

In this paper, we introduce a new, three-dimensional chaotic system with one stable equilibrium. This system is a multistable dynamic system in which the strange attractor is hidden. We investigate its dynamic properties through equilibrium analysis, a bifurcation diagram and Lyapunov exponents. Such multistable systems are important in engineering. We perform an entropy analysis, parameter estimation and circuit design using this new system to show its feasibility and ability to be used in engineering applications.

## 1. Introduction

Chaotic systems are very important in nonlinear dynamics. Many researchers are investigating the reason for the existence of chaotic attractors. For many years, researchers thought that the existence of a saddle equilibrium [1,2] is a necessary condition for strange attractors. However, in recent years many chaotic systems with no saddle point have been proposed. For example, we note systems with chaotic attractors and without any equilibria [3,4], with stable equilibria [5,6], with a line of equilibria [7,8], with a curve of equilibria [9,10], with circle and square equilibria [11], with a circular equilibria [12], with ellipsoid equilibria [13] and with a plane or surface of equilibria [14,15,16,17].

Leonov and Kusnetsov have introduced a new topic in nonlinear dynamics that has been called hidden attractors [18,19,20]. Hidden attractors are attractors in which the basin of attraction does not intersect with any equilibrium point [21,22,23]. The opposite side of this definition is self-excited attractors. A self-excited attractor has a basin of attraction that is associated with at least one unstable equilibrium [24,25,26]. Many unusual chaotic systems that have been proposed recently are systems with hidden attractors [27]. Hidden attractors in fractional order systems are also studied in [28,29,30].

Multistability is another important phenomenon that can be observed in dynamic systems [31,32,33]. In multistable systems, the final state of the system is dependent on the initial conditions [34,35]. Chaotic systems with stable equilibria are examples of multistable systems [36,37].

The quantification of chaotic attractors is a challenging topic in nonlinear dynamics. There are many measures that are used in this area. The main such measure is the Lyapunov exponent [38]. Entropy is another measure that determines the unpredictability of complex dynamics [39]. Entropy can be helpful in short time series [40], while the Lyapunov exponent is not suitable for them.

In this paper, a new three-dimensional chaotic flow with one stable equilibrium is proposed. The chaotic attractor of this system is hidden since it cannot be found using the stable equilibrium point. The rest of the paper is organized as follows:

The new chaotic system is proposed in Section 2. Some of its dynamic properties are investigated in Section 3. Section 4 discuses the complexity of the system’s attractors. Section 5 is devoted to the parameter estimation of the proposed system. The circuit implementation of the system is carried out in Section 6. Finally, the paper is concluded in Section 7.

## 2. System Description

In this paper, we are going investigate the dynamic properties of the following system:

(1) $$\dot{x}=z\;\dot{y}=-x-z\;\dot{z}=0.1x+5y-z+xy-0.3xz+a$$

where parameter a=1. System (1) is a three-dimensional chaotic flow that can have a chaotic attractor. This system has been designed based on the method proposed in [41]. In the first step of investigating its dynamic properties, the equilibrium points of the system were calculated. By setting zero at the right hand side of this equation we obtain:

(2) $$z=0\;x=0\;y=-\frac{1}{5}$$

Thus, the system has one equilibrium point in (0, −0.2, 0). A stability analysis of this equilibrium point can be carried out using the following Jacobian matrix at the equilibrium:

(3) $$J=\left[\begin{array}{ccc} 0 & 0 & 1\\ -1 & 0 & -1\\ -0.1 & 5 & -1 \end{array}\right]$$

By solving the equality det(λI−J)=0, the characteristic equation of System (1) is determined as follows:

(4) $$\lambda^{3}+\lambda^{2}+5.1\lambda +5=0$$

**Figure 1:**
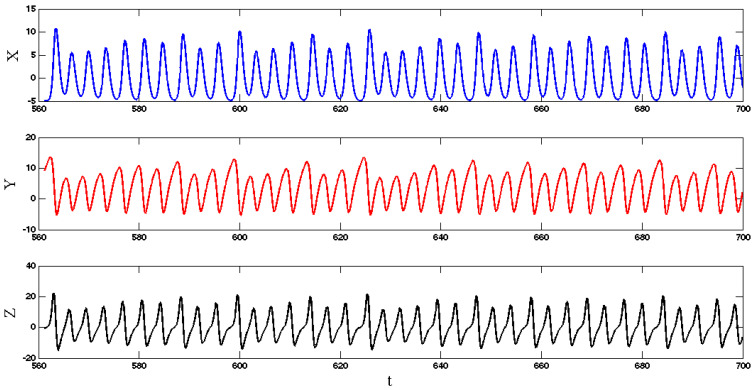
Time series of System (1) with parameter a=1 and initial conditions $\left(x_{0},y_{0},z_{0}\right)=\left(5.4,\;-1.8,\;3.3\right)$.

**Figure 2:**
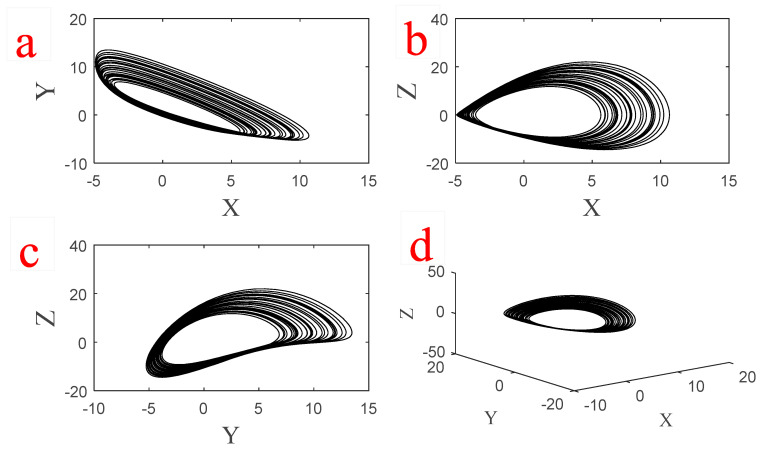
Three projections of the chaotic attractor of System (1) with parameter a=1 and initial conditions $\left(x_{0},y_{0},z_{0}\right)=\left(5.4,\;-1.8,\;3.3\right)$ in (**a**) *X-Y* plane. (**b**) *X-Z* plane. (**c**) *Y-Z* plane and (**d**) 3-D chaotic attractor.

**Figure 3:**
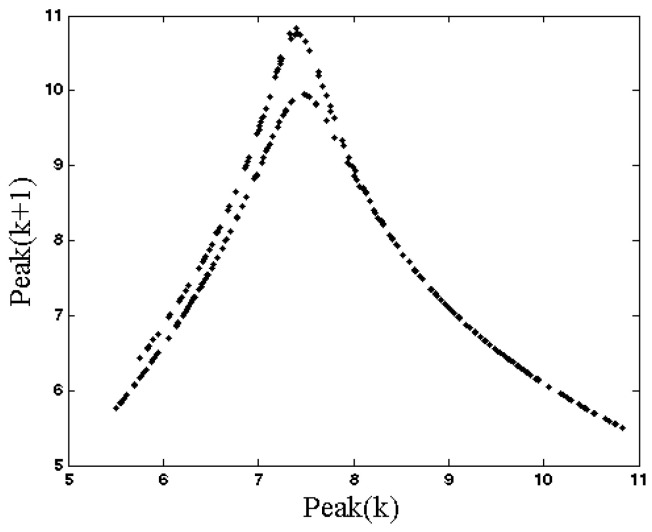
Poincaré map (peaks of *x* variable) of System (1) with parameter a=1 and initial conditions $\left(x_{0},y_{0},z_{0}\right)=\left(5.4,\;-1.8,\;3.3\right)$.

Solving Equation (4), we find that System (1) has three eigenvalues ($\lambda_{1}=-0.9835,\;\lambda_{2,3}=-0.0082\pm 2.2547i$) for the equilibrium (0, −0.2, 0). Thus, it is a stable equilibrium point. Every other possible attractor of this system coexists with this stable equilibrium point. The system shows a chaotic attractor if we choose initial conditions $\left(x_{0},y_{0},z_{0}\right)=\left(5.4,\;-1.8,\;3.3\right)$. The chaotic attractor cannot be found using any equilibrium points of the system since the system has only one stable equilibrium point. Thus the strange attractor is hidden [27]. The time series of three states of System (1) for a=1 are shown in Figure 1. Three projections of the chaotic attractor and its three-dimensional attractor are presented in Figure 2 and its Poincaré map is shown in Figure 3. In this plot, we use the peak values of x variable as the Poincaré map.

## 3. Bifurcation Analysis

**Figure 4:**
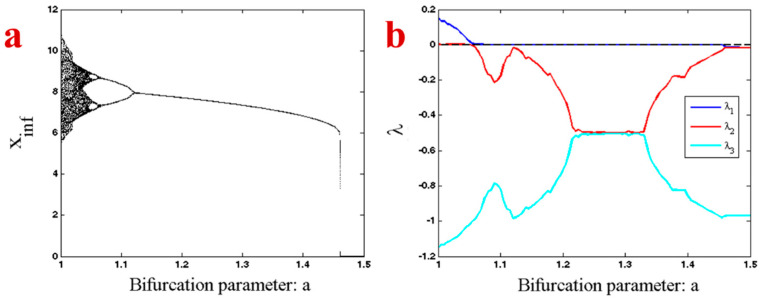
(**a**) Bifurcation diagram of System (1) with respect to the changing parameter *a* in the interval [1, 1.5] and forward continuation. (**b**) Lyapunov exponents of System (1) with respect to the changing parameter *a* in the interval [1, 1.5] and forward continuation.

In order to show the different dynamic behaviors of System (1), its bifurcation diagram was investigated. Figure 4a shows a bifurcation diagram of the system with respect to the changing parameter a. The system has an inverse route of period doubling after its chaotic behavior. The dynamic of the system also suddenly changes in a=1.46 from a stable limit cycle to a stable equilibrium. In other words, System (1) has a chaotic attractor in parameter a=1 and initial conditions $\left(x_{0},y_{0},z_{0}\right)=\left(5.4,\;-1.8,\;3.3\right)$. The system also has a stable equilibrium point in $\left(0,-\frac{a}{5},0\right)=\left(0,-\frac{1}{5},0\right)$. Thus, there are some initial conditions in the vicinity of this stable equilibrium that are attracted to it. In the bifurcation diagram of Figure 4, we used the initial conditions $\left(x_{0},y_{0},z_{0}\right)=\left(5.4,\;-1.8,\;3.3\right)$ for parameter a=1 and applied the forward continuation method for the higher values of parameter a. In other words, in the higher values of parameter a, we used initial conditions from the end of trajectory in the previous parameter with forward changing. Thus, the trajectory of the system traps into one attractor that is chaotic in parameter a=1 and bifurcates with an inverse route of period doubling to chaos. In parameter a=1.46 the previous attractor becomes unstable and the system jumps from a stable limit cycle to the stable equilibrium point $\left(0,-\frac{a}{5},0\right)$. To be sure about the chaotic and other types of attractors of the system, it was necessary to calculate the Lyapunov exponents (Figure 4b). In the smaller values of the parameter a the system has a chaotic attractor (one positive, one zero, and one negative LE). It then shows the limit cycle, since its largest Lyapunov exponent is zero and the other two LEs are negative. After that, in the higher values of parameter a, the attractor changed to a stable equilibrium that has three negative Lyapunov exponents.

## 4. Entropy Analysis

Entropy is a measure of unpredictability. Shannon has proposed a formulation for calculating entropy [42]. Since chaotic attractors have an infinite number of states, another type of entropy is needed to calculate their unpredictability. This entropy is called Kolmogorov–Sinai ($H_{ks}$) [40,43] and its formulation is shown in Equation (5).

(5) $$H_{ks}\left(\beta \left[\varepsilon \right]\right)=\frac{1}{\tau_{min}\left(\beta \left[\varepsilon \right]\right)}\underset{\tau }{\sum }\rho \left(\tau ,\beta \left[\varepsilon \right]\right)log\left(\frac{1}{\rho \left(\tau ,\beta \left[\varepsilon \right]\right)}\right)$$

**Figure 5:**
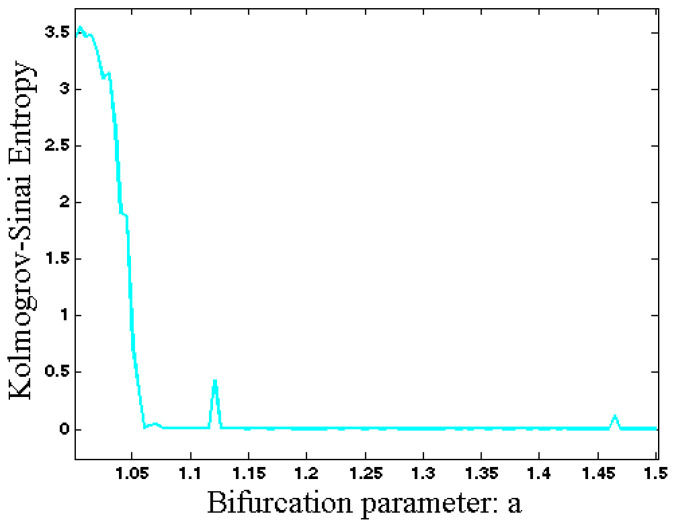
Kolmogorov–Sinai entropy of System (1) with respect to changing parameter *a*.

It is defined using the first Poincaré recurrence times (FPRs) denoted by $\tau_{i}$. β is a D-dimensional box in the state space with side $\varepsilon_{1}$ where the FPRs are observed. ρ(τ,β) is the probability distribution of $\tau_{i}$. For a smooth chaotic system $H_{ks}$ is equal to the sum of all positive Lyapunov exponents [44,45]. The Kolmogorov–Sinai entropy of System (1) with respect to the changing parameter a is shown in Figure 5. Near a bifurcation point, the system’s state becomes slower. In other words, the transient time increases near a bifurcation point [46,47]. In order to use Kolmogorov–Sinai entropy to anticipate a bifurcation point, we calculated it without removing the transient time of the trajectory. If we remove the transient time, then the estimated Kolmogorov–Sinai entropy became zero in regular dynamics and it changed through variations in the final state of the system. By applying Kolmogorov–Sinai entropy to the system’s state without removing transient time, we were able to see complexity of transient parts as well as final state of the system. As Figure 5 shows, in small values of parameter a the system has a chaotic attractor and its unpredictability is high. By increasing parameter a, the system changes its dynamic to a regular dynamic and thus its entropy decreases. However, in the bifurcation points the system becomes slower and its transient time increases. That is the reason for the increasing entropy in the bifurcation points.

## 5. Parameter Estimation

**Figure 6:**
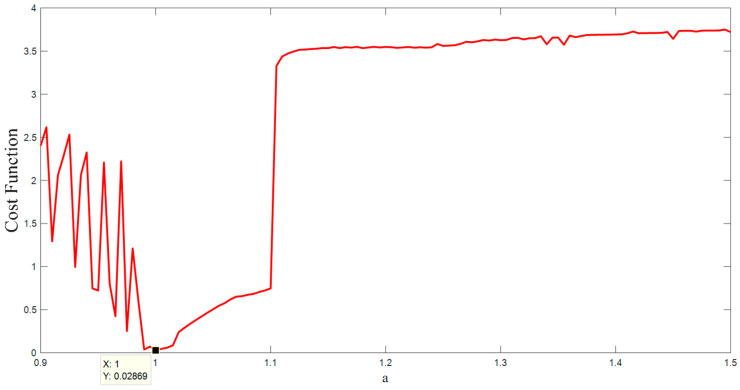
The value of the cost function with respect to changing the parameter *a*.

**Figure 7:**
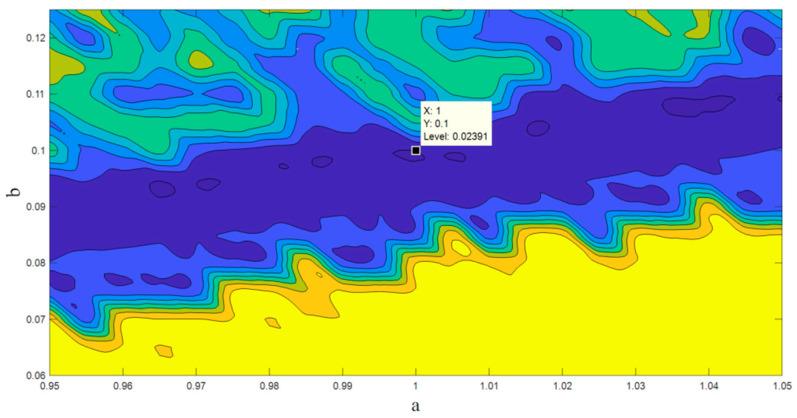
The value of the cost function with respect to changing the parameter *a* & *b*.

There are various methods for parameter estimation in dynamic systems that are based on optimization methods [48,49,50]. The basis of these methods is a cost function associated with the differences between the time series obtained from a real system and the time series obtained from a known model with unknown parameters. However, these approaches are not appropriate for chaotic systems due to the butterfly effect [51,52,53]. Therefore an alternative method is proposed in [54,55]. This new method changes the analyzing domain of the chaotic system from time space to the state space. In the other words, this new model compares the topology and structure of the points in the state space. To this end, the algorithm searches the space of the parameter to find the most similar point from the model to the point of the data. Whenever the structure of the points in the state space gets close enough to the structure of the real data, the optimum parameter is found. For more complete details, see [55,56]. In this paper, we used this useful cost function along with WOA (whale optimization algorithm [57,58]) for the parameter estimation method. Figure 6 shows the result of the cost function with respect to changing the parameter a. As is shown in the figure, the global minimum is located exactly in the main parameter a=1. Figure 7 shows the result of the cost function with respect to changing the parameters a and *b* (consider *b* as the coefficient of x in the third equation of Equation (1)). As is shown in the figure, the global minimum is located in the main parameters a=1 & b=0.1.

One of the most efficient categories of the optimization methods is meta-heuristic methods, which cover a wide range of problems, especially in engineering applications [50,59,60,61]. Most of them are inspired by nature. Humpback whales’ hunting behavior in sea form the basis of the WOA (whale optimization algorithm) meta-heuristic algorithm [57,58]. The hunting behavior of Humpback whales, who encircle the recognized location of prey, has become the basis of the WOA algorithm. In this algorithm the target prey is the current best candidate or close to the optimum solution and the attacking strategy is a bubble-net strategy. By considering all these together, the WOA optimization method can be explained through three steps: Finding the prey, encircling the prey, and the bubble-net attacking behavior of humpback whales.

**Figure 8:**
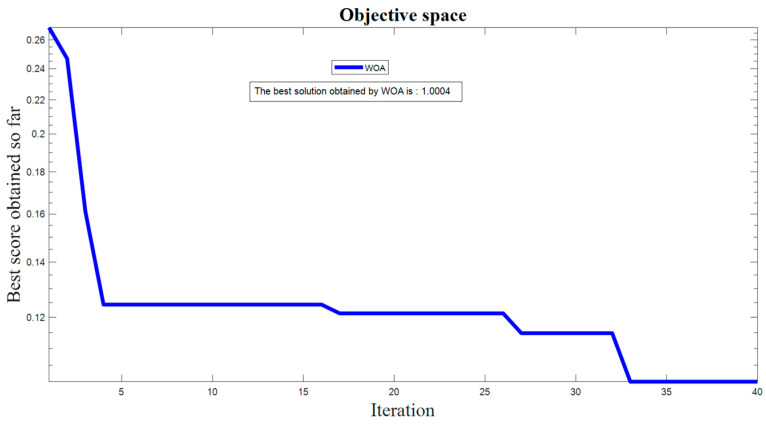
The result of WOA for the 30 searching agents and 40 iterations.

At first, the algorithm determines the best candidate solution. Then it updates the position of the other points in order to get closer to the best agent. The second step is about the attack strategy, which can be divided into two approaches. The first is a shrinking encircling mechanism and the other is a spiral updating position. For complete details see [57]. Figure 8 represents the result of the WOA for the 30 searching agents and 40 iterations.

## 6. Circuit Design

**Figure 9:**
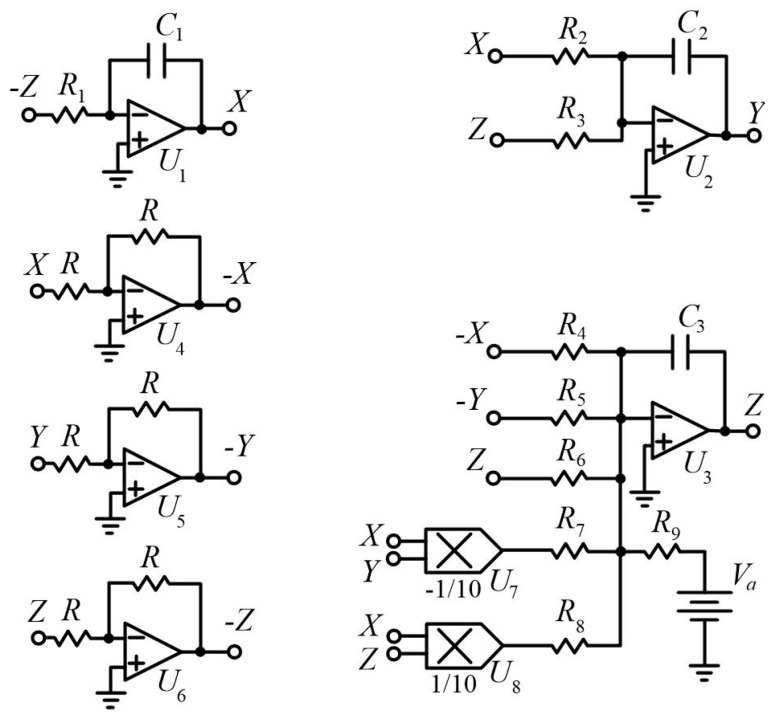
The circuit constructed by six operational amplifiers $\left(U_{1}-U_{6}\right)$ and electronic elements.

This section presents a circuit implementation for the three-dimensional flow (1) (see Figure 9). The circuit implementation in Figure 9 was constructed using six operational amplifiers $\left(U_{1}-U_{6}\right)$ and electronic elements [62,63,64,65,66]. We used TL084 operational amplifiers and AD633 analog multipliers. Taking the voltages of three operational amplifiers $\left(U_{1},\;U_{2},\;U_{3}\right)$ as X, Y, Z, it confirms that the circuit in Equation (6) corresponds to the flow (1):

(6) $$\dot{X}=\frac{1}{R_{1}C_{1}}Z\;\dot{Y}=-\frac{1}{R_{2}C_{2}}X-\frac{1}{R_{3}C_{2}}Z\;\dot{Z}=\frac{1}{R_{4}C_{3}}X+\frac{1}{R_{5}C_{3}}Y-\frac{1}{R_{6}C_{3}}Z+\frac{1}{R_{7}C_{3}10V}XY-\frac{1}{R_{8}C_{3}10V}XZ-\frac{1}{R_{9}C_{3}}V_{a}$$

where $V_{a}$ is a DC voltage source.

**Figure 10:**
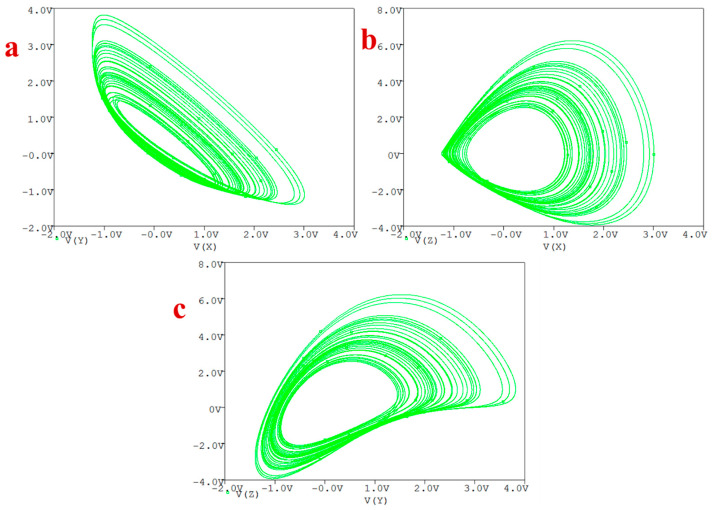
Generated attractors in PSpice of the circuit: (**a**) *X*-*Y* plane, (**b**) *X*-*Z* plane, (**c**) *Y*-*Z* plane.

The circuit generates chaos as illustrated in Figure 10 for the following set of components: $C_{1}=C_{2}=C_{3}=20\;\mathrm{nF}$, $R_{1}=R_{2}=R_{3}=R_{6}=R=40\;k\Omega$, $R_{4}=400\;k\Omega$, $R_{5}=8\;k\Omega$, $R_{7}=1\;k\Omega$, $R_{8}=3.333\;k\Omega$, $R_{9}=160\;k\Omega$, and $V_{a}=-1V_{DC}$.

## 7. Conclusions

A new three-dimensional chaotic system with one stable equilibrium was proposed in this paper. A bifurcation analysis of the system showed an inverse period doubling route to chaos with respect to increasing parameter a. The unpredictability of its dynamic was discussed using Kolmogorov–Sinai entropy. Parameter estimation of the system was carried out and circuit implementation of the system confirmed its feasibility. It is noted that the real practical realization and real laboratory measurements of the circuit should be carried out. Therefore, practical results will be reported in our next works.
